# Biomimetic Layer-by-Layer Self-Assembly of Nanofilms, Nanocoatings, and 3D Scaffolds for Tissue Engineering

**DOI:** 10.3390/ijms19061641

**Published:** 2018-06-01

**Authors:** Shichao Zhang, Malcolm Xing, Bingyun Li

**Affiliations:** 1 Department of Orthopaedics, School of Medicine, West Virginia University, Morgantown, WV 26506, USA; Shichao.zhang@hsc.wvu.edu; 2 Department of Mechanical Engineering, University of Manitoba, Winnipeg, MB R3T 2N2, Canada; Malcolm.Xing@umanitoba.ca; 3 The Children’s Hospital Research Institute of Manitoba, Winnipeg, MB R3E 3P4, Canada; 4 West Virginia University Cancer Institute, Morgantown, WV 26506, USA

**Keywords:** layer-by-layer, self-assembly, polyelectrolyte, multilayer, nanofilm, nanocoating, biomaterial, scaffold, tissue engineering

## Abstract

Achieving surface design and control of biomaterial scaffolds with nanometer- or micrometer-scaled functional films is critical to mimic the unique features of native extracellular matrices, which has significant technological implications for tissue engineering including cell-seeded scaffolds, microbioreactors, cell assembly, tissue regeneration, etc. Compared with other techniques available for surface design, layer-by-layer (LbL) self-assembly technology has attracted extensive attention because of its integrated features of simplicity, versatility, and nanoscale control. Here we present a brief overview of current state-of-the-art research related to the LbL self-assembly technique and its assembled biomaterials as scaffolds for tissue engineering. An overview of the LbL self-assembly technique, with a focus on issues associated with distinct routes and driving forces of self-assembly, is described briefly. Then, we highlight the controllable fabrication, properties, and applications of LbL self-assembly biomaterials in the forms of multilayer nanofilms, scaffold nanocoatings, and three-dimensional scaffolds to systematically demonstrate advances in LbL self-assembly in the field of tissue engineering. LbL self-assembly not only provides advances for molecular deposition but also opens avenues for the design and development of innovative biomaterials for tissue engineering.

## 1. Introduction

Tissue engineering, an interdisciplinary area that evolved from biomaterials and engineering development, aims to assemble scaffolds, cells, and functional molecules into tissues to create biological alternatives for damaged tissues or organs [1,2,3,4,5]. To obtain the desired outcomes, biomaterial scaffolds that act as templates for tissue regeneration should induce appropriate cellular responses, guide the growth of new functional tissues, and support organ systems. To mimic the extracellular matrix (ECM) of native tissues, the ideal scaffolds must meet some specific requirements, involving structural, physical, chemical, and biological properties and functions [6,7,8]. Among these requirements, the surface properties of the scaffolds have long been recognized as being of utmost importance due to the direct interface between materials and cells as well as tissues [9,10,11]. Therefore, one major challenge in tissue engineering is to control the surface properties of biomaterials (especially at the molecular level), to modify the behavior of cells, and to tune the formation of new tissues.

**Figure 1:**
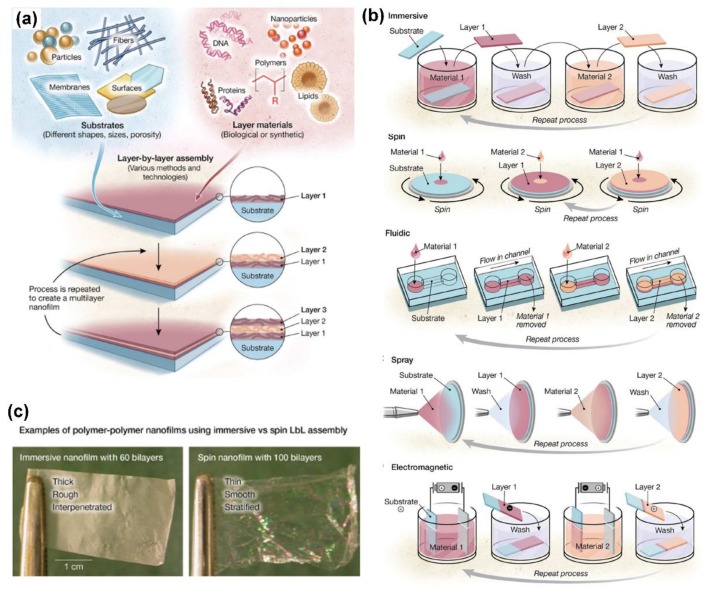
(**a**) Schematic overview of LbL assembly technique with fabrication capacity on any type of substrates and from an extensive choice of materials; (**b**) schematics showing the five main technology categories for LbL assembly; (**c**) a typical comparison of different films using immersive and spin assembly. Reprinted from [17] with permission from American Association for the Advancement of Science.

Up to now, considerable efforts have been devoted to functionalizing biomaterial surfaces for tissue engineering. Due to the ability to regulate the assembly of coating at the nanometer- or micrometer-scale, self-assembled monolayer assembly and Langmuir-Blodgett deposition have shown remarkable capability in modifying material surfaces for tissue engineering applications [12,13,14,15,16]. However, several intrinsic limitations of these two techniques, including a long fabrication period, limited raw material types, low formation efficiency, limited stability, and expensive instrumentation, have restricted their practical applications. In contrast, layer-by-layer (LbL) assembly is a highly versatile and simple multilayer self-assembly technique; it has the capability to fabricate multilayer coatings with controlled architectures and compositions from extensive choices of usable materials for various biomedical applications (as shown in Figure 1a) [17,18,19,20,21,22,23].

Benefiting from different driving forces and assembly technologies of LbL self-assembly, various LbL assembly biomaterials have been prepared from different material species, including polyelectrolytes, biomolecules, colloids, particles, etc., and have shown remarkable physical, chemical, and biological properties/functions in the field of tissue engineering [20,24,25]. In this review, we attempt to present a brief overview of recent advances in designing and fabricating LbL self-assembly biomaterial scaffolds for tissue engineering applications. The advanced LbL assembly technique is introduced, ranging from origin, technology, and mechanisms to biomedical applications. Furthermore, we highlight recent advances in controllable fabrication, properties, and performance of LbL assembly in the forms of multilayer nanofilms, nanocoatings, and three-dimensional (3D) scaffolds for tissue engineering. Finally, we discuss the perspectives of further research directions in the development of LbL assembly for tissue engineering.

## 2. LbL Self-Assembly Technology

### 2.1. Origin and Definition

LbL assembly is an alternative to self-assembled monolayer assembly and Langmuir-Blodgett deposition, which are two dominant techniques for obtaining solid films at the molecular level. LbL assembly was first proposed by Iler in 1966 and achieved substantial development after the pioneering work of Decher et al. in the 1990s [18,20,26]. Since then, LbL assembly technique has become an efficient, facile, flexible, and versatile strategy to coat substrates with multilayers of controlled structures, properties, and functions for various applications [26]. The process of LbL assembly is simple and can be accurately controlled to create finely tailored structures [27,28]. Typically, the LbL assembly process includes the sequential adsorption of complementary molecules on a substrate surface, driven by multiple interactions involving electrostatic and/or nonelectrostatic interactions. Between the adsorption steps for each layer deposition, steps of washing and drying are usually introduced to avoid contamination of the next solution due to liquid adhering on substrates from the former solution, and to elute the loose molecules and stabilize them in the formed layers. These deposition and wash steps can be repeated to achieve the desired number of deposition layers. Moreover, fine control of composition, thickness, and topography can be achieved by adjusting the assembly parameters involving solution properties, like concentration, ionic strength, and pH, and process parameters, such as temperature, time, and drying conditions [18,29,30,31,32,33,34]. Various building blocks used for LbL assembly include, but are not limited to, natural polymers, synthetic polymers, peptides, clays, metal oxides, polymer gels, and complexes of such materials [20,29,35,36,37]. Compared to other methods for fabricating nanofilms, there are three prominent advantages of the LbL assembly technique, these include precise control of the composition and structure of nanofilms, large-scale fabrication capacity on various types of substrates regardless of size and shape, and mild and confined formation environments.

### 2.2. Technology Categories and Mechanisms

The widespread use of LbL assembly for various applications with different processing requirements and tools has led to the design and development of a variety of deposition technologies, for instance, dipping, centrifugation, roll-to-roll, calculated saturation, creaming, immobilization, atomization, spinning, spraying, magnetic assembly, high gravity, electrodeposition, electrocoupling, filtration, fluidics, and fluidized beds [17,38,39,40,41,42,43]. These technologies can be divided into five main categories, including (i) immersion; (ii) spin; (iii) spray; (iv) electromagnetic driven; and (v) fluidic assembly, as shown in Figure 1b. Taking into account the diversity of process properties and resulted nanofilm properties due to the assembly technologies, the proper choice of assembly technology is crucial for controllable fabrication and successful application of the assembled nanostructured materials. Different assembly methods usually result in different structures and properties of the assembled materials.

Immersive LbL assembly, also called dip assembly, including different techniques like dewetting, roll-to-roll, centrifugation, creaming, and so on, is the most widely used method and can form interpenetrated layered structures [17,44]. Immersive assembly allows for nanofilms on substrates of almost any shape or size. The challenge of this technique is to achieve reduced assembly times and to create automated systems with less manual intervention, which has attracted much attention and research. LbL spin assembly employs the common coating technology of spinning surface to construct nanostructured materials [17]. It usually results in humongous nanofilms with a short assembly process; however, it is limited to coating small planar substrates, as shown in Figure 1c. LbL assembly using spray coating can form films based on aerosolizing solutions and facilely spraying aerosols onto planar materials and particulates [45]. There are two main forces governing spray assembly: Bulk movement in the spray and random movement in the liquid film. Due to the unique manner, spray assembly can fabricate films on industrial-scale substrates with any surface topography and the resultant films usually show distinct layered structures. Electromagnetic assembly is a relatively new method based on applied electric or magnetic field to form layered structures, showing a substantially different driving force, like current-induced pH change and redox-reactions [43,46]. Although this technology usually requires special equipment and expertise, it does provide a new strategy for multilayer film assembly. By coating channel walls or substrates placed in channels, fluidic assembly technique is proposed to perform complicated assembly on designed 3D structures and surfaces that are not easily accessible to other technologies, providing a novel strategy for region-specific patterning and low reagent consumption [5,47]. The general method involves using vacuum or pressure to move polymer and washing solutions through the channels, like capillaries, tubing, microfluidic networks, etc. Especially, besides planar substrates, 3D aerogels and small particles (<5 μm) can also be coated using fluidic assembly, which can function as a valuable tool to coat-sensitive particulate substrates. In despite of the required special equipment and operation, fluidic assembly greatly increases the industrial capacity of multilayer assemblies by using various substrates along with reduced reagent consumption.

**Figure 2:**
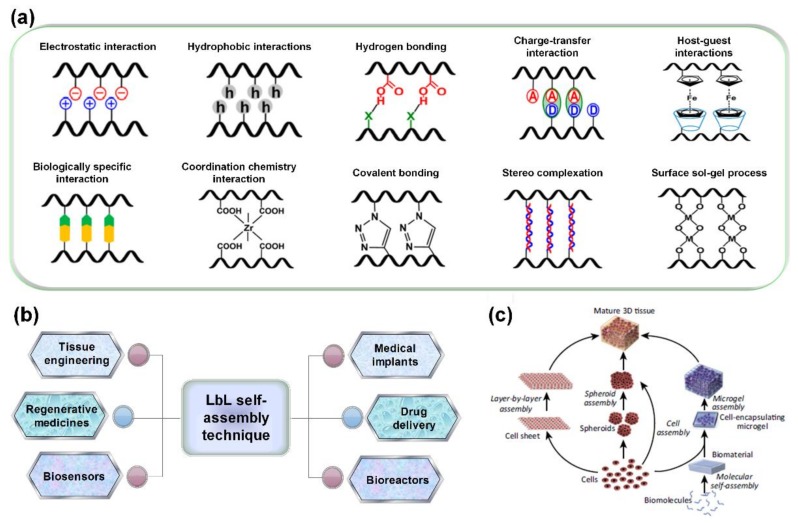
(**a**) Molecular interactions driving the LbL self-assembly of materials; (**b**) schematic showing the main biomedical application fields for LbL self-assembly technique; (**c**) schematic of multiscale assembly strategies for engineering tissue constructs. Reprinted from [18,56] with permissions from American Chemical Society and Elsevier.

Conventionally, electrostatic interaction is the primary and most widely used driving force for the formation of nanostructured multilayer films, as presented in Figure 2a. Besides electrostatic interaction, LbL assembly can be driven by multiple other interactions, including hydrogen bonds, halogen bonds, charge-transfer interactions, covalent bonds, host-guest interactions, coordination chemistry interactions, biologically specific interactions, stereo complexation, surface sol-gel process, etc. [18]. Each of these intermolecular interactions has advantages and disadvantages. The driving forces and combinations among them can be utilized to significantly enrich the fabrication and application of LbL assembly materials for tissue engineering and biomedicine, bioelectronics, drug delivery, environment, energy, and information storage [48,49,50].

### 2.3. Biomedical Applications

The LbL self-assembly technique has been widely used for various biomedical applications including tissue engineering, medical implants, regenerative medicines, drug delivery, biosensors, bioreactors, and so on (Figure 2b) [35,51,52,53,54,55]. The capacity to manipulate the chemical, physical, and topographical properties of nanostructured architectures by facilely adjusting solution properties and assembly parameters, not only allows LbL self-assembly to be suitable to investigate the effects of external stimuli on cellular responsiveness but also provides a new strategy for creating two/three-dimensional (2/3D) nanostructured architectures and coatings for individual cells or scaffolds for tissue engineering (Figure 2c) [56,57,58,59]. The uses of LbL self-assembly biomaterials include nanostructured coating or materials to either promote or prevent cell adhesion, to maintain and direct cellular phenotypes, and to provide 3D scaffolds for cell culture or co-culture. Since the other biomedical applications of LbL assembled materials have been extensively discussed and reviewed, this review will focus on the modulation of structures and functions, and applications of LbL assembly in the field of tissue engineering.

## 3. LbL Self-Assembly of 2D Multilayer Nanofilms for Tissue Engineering

### 3.1. LbL Multilayer Nanofilms Directing Cellular Phenotypes

In biology, ECM is a collection of extracellular molecules secreted by cells that provide structural and biochemical support to the surrounding cells [60]. Taking into account the key role of ECM, various ECM molecules like collagen, polysaccharide, etc. have been used to fabricate multilayer nanofilms for tissue engineering using the LbL self-assembly technique [61,62,63,64,65]. Among these ECM molecules, collagen is most widely used in combination with other natural polyelectrolytes. The structures and properties involving surface morphology, thickness, zeta potential, surface roughness, and cellular phenotype like cell adhesion, cell growth, and cell differentiation have been investigated to demonstrate the feasibility of designing functional biomaterials by means of LbL assembly. For instance, Wittmer et al. fabricated various multilayer nanofilms composed of polysaccharides, polypeptides, and synthetic polymers, and investigated the adhesion and function of various hepatic cells in terms of terminal layer, film composition, charge, rigidity, and presence of biofunctional species [65]. This study offered the key variables in promoting attachment and function of hepatic cells and provided a promising candidate for in vivo human liver tissue engineering applications.

**Figure 3:**
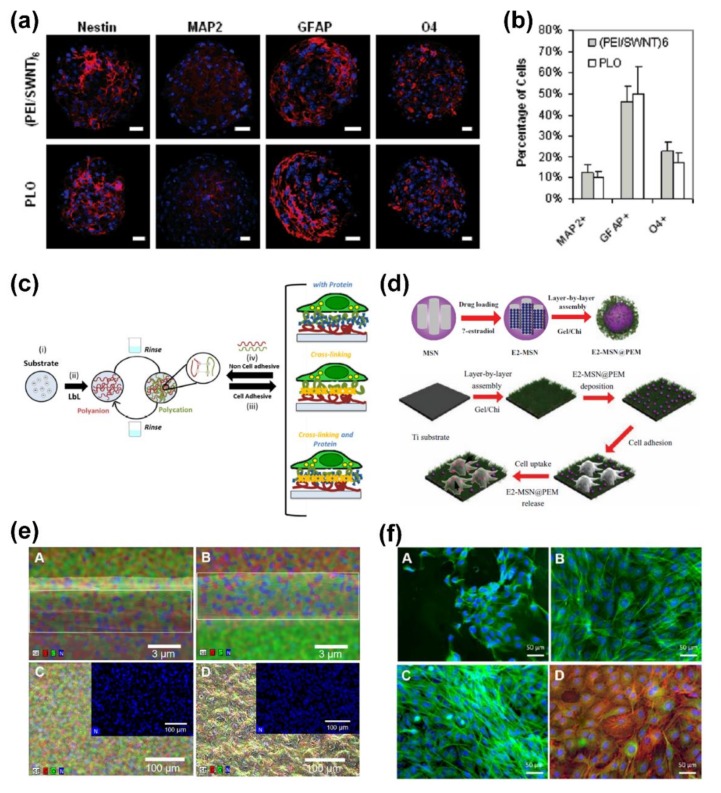
(**a**) Confocal microscopy images of differentiated neurospheres on day 7; (**b**) average percentages of differentiated cell phenotypes after 7 days in culture; (**c**) schematic showing LbL assembly of polyelectrolytes based on electrostatic interactions for tuning cell adhesive properties using cross-linking; (**d**) schematic illustration of the fabrication and cell uptake of LbL assembly multilayers embedded with β-estradiol-silica nanoparticles onto substrates; (**e**) mixed element map of the cross-section and upper surface of different catechol-based freestanding membranes; (**f**) Osteopontin immunofluorescence images of cells after 14 days cultured on different catechol-based freestanding membranes. Reprinted from [24,72,77] with permissions from American Chemical Society, Elsevier, and Wiley-VCH.

Single-walled carbon nanotubes (SWNTs) can not only exhibit a series of unique mechanical and electrical properties, but also demonstrate fascinating cell effect due to their electrical stimulation for neurological- or brain-related tissue engineering. Gheith et al. prepared SWNT multilayer films using charged nanotubes coated with designed copolymers, which exhibited high electrical conductivity to electrically stimulate excitable neuronal cells [66]. The resultant assembly films showed a clear demonstration of electrical excitation of neurons when a current was passed through the LbL films, revealing the capacity to adjust the biological activity of films and their interaction with cells. SWNT/polyelectrolyte multilayer films were also fabricated by Jan et al., on which environment-sensitive embryonic neural stem cells from the cortex were successfully differentiated into neurons, astrocytes, and oligodendrocytes, as shown in Figure 3a,b [24].

Up to now, considerable efforts have been devoted to optimizing the surface properties of LbL assembly films, such as rigidity, roughness, hydrophilicity, etc. [67,68,69,70]. Among them, cross-linking of layer components is the most widely used strategy. Hillberg et al. studied the effect of genipin cross-linking on the cellular adhesion properties of LbL polyelectrolyte films, and found that the cross-linking resulted in an increased cell adhesion and spreading on polymeric films [71]. They mainly focused on the investigation of film rigidity and cell adhesion of nanofilms with and without crossing-linking, providing an effective strategy for improving cell adhesion on nanofilms. However, no further detailed studies on surface chemistry of films and its effect on cell proliferation were carried out. By employing chitosan with/without cross-linking and/or coating with alginate, Silva et al. proposed a strategy to adjust the cell adhesion of LbL assembly films, as demonstrated in Figure 3c. The significant changes observed in cell adhesion, spreading, and proliferation can be attributed to the change of surface chemistry and mechanical properties due to cross-linking of multilayer films [72].

To further enhance the function of LbL assembly nanofilms, various functional fillers including nanoparticles, growth factors, and antibacterial agents, have been introduced to construct multilayer films as layer component and loading drugs [73,74,75,76,77,78,79]. For example, Hu and co-authors reported a strategy to construct hybrid chitosan/gelatin multilayers embedded with mesoporous silica nanoparticles on a titanium implant to regulate biological behavior of osteoblasts/osteoclasts in vitro, as presented in Figure 3d [77]. In addition, the free-standing multilayer nanofilms composed of chitosan and dopamine-modified hyaluronic acid were prepared by Sousa et al., and exhibited enhanced cell adhesion, viability, and proliferation for bone tissue engineering by optimizing their morphology, chemistry, and mechanical properties (Figure 3e,f) [80].

### 3.2. LbL Multilayer Nanofilms Encapsulating Cells and Tissues

**Figure 4:**
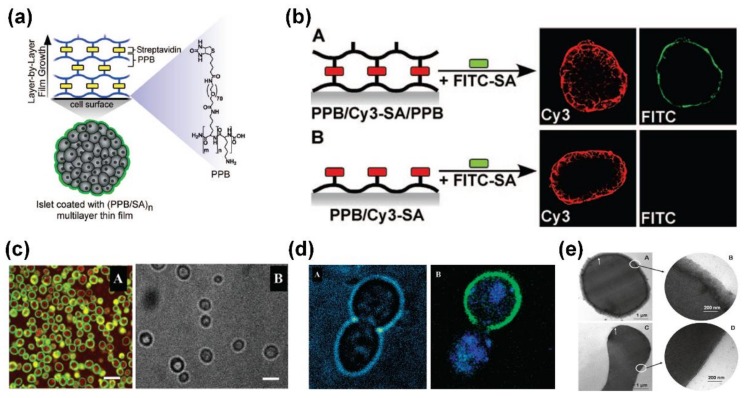
(**a**) Schematic illustration of the fabrication of PEG-rich, nanothin conformal islet nanofilms via LbL assembly; (**b**) Poly(l-lysine)-*g*-poly(ethylene glycol)(biotin)/streptavidin multilayer films assembled on individual pancreatic islets; (**c**) confocal images of freshly coated living cells and (**d**) transmission image of cells during their duplicating process; (**e**) transmission electron microscope images of coated and uncoated red blood cells with LbL assembly films. Reprinted from [82,84,85] with permissions from American Chemical Society.

Encapsulation of live cells and tissue offers an effective strategy to modulate cells or tune the response to their environment, especially for attenuating deleterious host responses toward transplanted cells [70,81]. Because of its ability to generate films of nanometer thickness on chemically and geometrically diverse substrates under mild formation environments, LbL assembly has emerged as an ideal technique compared to other methods, and can greatly minimize transplant volume for cell/tissue encapsulation in the field of tissue engineering. Wilson et al. reported the successful intraportal islet transplantation by LbL self-assembly of poly(l-lysine)-*g*-poly(ethylene glycol) (biotin) and streptavidin, and the resulted nanothin; PEG-rich conformal coatings can be tuned to coat inlets without loss of their viability and function, revealing a unique approach to modify the biochemical surfaces of living cells and tissues for various tissue engineering applications, as shown in Figure 4a,b [82]. Single living cell encapsulation in nano-organized polyelectrolyte shells were reported and the resultant shells could effectively protect cell integrity and preserve cell metabolic activities (Figure 4c,d) [83,84]. Recently, Mansouri et al. prepared nonimmunogenic polyelectrolyte multilayer films composed with alginate, chitosan-graft-phosphorylcholine and poly-l-lysine-graft-polyethylene glycol using LbL assembly; they investigated these nanofilms on fully functional human red blood cells in suspension for attenuated immune response, as exhibited in Figure 4e [85]. The resultant nonimmunogenic films not only create an advanced nanomaterial for production of universal red blood cells, but also provide an effective strategy for the design and development of functional multilayers for cell and tissue encapsulation.

## 4. LbL Self-Assembly of Scaffold Nanocoatings for Tissue Engineering

### 4.1. LbL Scaffold Coating Directing Cellular Phenotypes

To engineer tissues in vitro, cells are usually cultured on 3D scaffolds that provide the bioactive cues to guide their growth and differentiation into tissues. Designing the physical and chemical properties of scaffold surfaces has become a crucial aspect for tissue engineering as it can directly tune the cell responses to 3D scaffolds. Among various methods for modifying scaffold surfaces, LbL assembly is highly attractive due to its capability to conformally coat complicated geometries and its tunability of incorporation. Due to their good biocompatibility, ECM components including collagen, chitosan, and gelatin have been widely used to construct scaffold nanocoating using LbL assembly [86,87,88,89,90,91]. For example, the poly(styrene sulfonate)/chitosan multilayer films were prepared to modify poly(l-lactic acid) scaffold surfaces toward improving the scaffold cytocompatibility to human endothelial cells [86]. Various nanocoatings composed with positively charged macromolecules and negatively charged gelatin have been prepared to improve the surface biocompatibility of different tissue scaffolds, like titanium films, poly(d,l-lactide) films, poly(l-lactic acid) fibrous scaffolds, etc., and to promote cell adhesion, growth, and differentiation by modifying the structural and chemical properties of scaffolds [87,88,89]. For cartilage tissue engineering applications, collagen and chondroitin sulfate were deposited alternately on 3D scaffolds to optimize the cell-material interaction for enhancing chondrogenesis [90,91].

**Figure 5:**
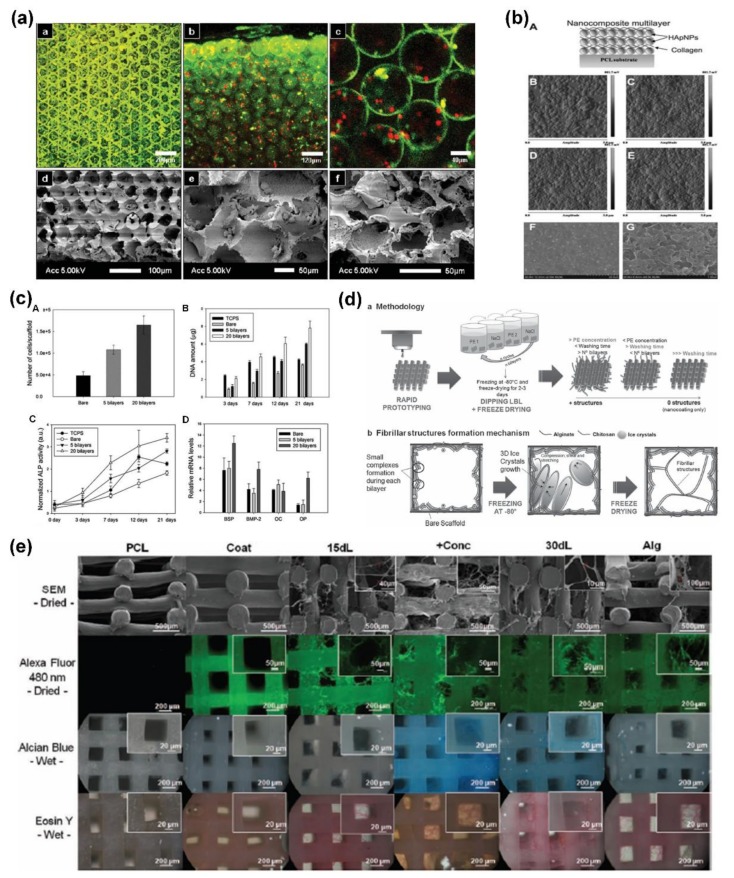
(**a**) Confocal images of inverted colloidal crystal scaffolds cultured with thymic epithelial cells and monocyte cells; (**b**) illustration of LbL multilayer nanocomposite coating with hydroxyapatite and collagen on substrates, and the AFM images of multilayers with different numbers of bilayers; (**c**) hMSCs adhesion and their quantification of DNA amounts to bare and coated scaffolds, and alkaline phosphatase activity and relative mRNA expression during the culture of hMSCs on various substrates; (**d**) steps and mechanism for developing the hierarchical and hybrid 3D scaffolds and (**e**) representative images of the structures of these scaffolds. Reprinted from [49,92,94] with permissions from Wiley-VCH and Royal Society of Chemistry.

Besides the ECM components, biocompatible inorganic materials, such as clay, calcium phosphate, and hydroxyapatite have been used as building blocks to modify 3D scaffold surfaces using LbL assembly. Lee and co-workers prepared new LbL assembly of clay/poly(diallyldimethylammonium chloride) multilayers on inverted colloidal crystal scaffolds, and examined their effect on cell adhesion and differentiation from both experimental and modeling aspects (Figure 5a) [92]. This study not only created new nanofilm-coated materials as 3D microenvironments for cellular co-cultures, but also provided a strategy to efficiently simulate differentiation niches for the different components of hematopoietic systems. An electrospun fiber scaffold coated with LbL assembly of gelatin and calcium phosphate films was prepared by Li et al. for bone tissue engineering [93]. This modified scaffold could effectively mimic the structure, composition, and biological function of the bone extracellular matrix, resulting in a significantly higher cell proliferation rate due to the mineralized nanocoating. Hydroxyapatite-based LbL assembly nanocoatings were also developed and used to enhance the osteogenic capacity of human mesenchymal stem cells (hMSCs) by providing additional bioactive agents, as shown in Figure 5b,c [94].

Recently, many non-conventional LbL self-assembly techniques and hierarchical structures incorporated with multi-functions have been proposed to design and develop novel scaffold nanocoatings for tissue engineering. Oliveira et al. reported a new approach to develop inner structures inside 3D scaffolds for tissue engineering [49]. By combining the non-conventional LbL assembly having incomplete washing steps with ice crystal growth, hierarchical and fibrillar structures could be created in the interior of 3D scaffolds, which could effectively enhance the surface area available for cell growth and mimic the natural environment of fibrillar extracellular matrix, as shown in Figure 5d,e. Spray-assisted LbL assembly was also proposed to fabricate hyaluronic acid and poly-l-lysine multilayers on porous hyaluronic acid scaffold for the development of a single epidermal-dermal scaffold to treat full-thickness skin defects [95]. In addition, various growth factors and antibacterial agents were also immobilized on 3D scaffolds to form mirco/nano-hierarchical structures using the LbL assembly technique [23,29,79,96]. The effective combination of hierarchical structures and incorporated fillers may result in multi-functions for various tissue engineering, especially for bone regeneration and anti-infection.

### 4.2. LbL Scaffold Coating for Cell Co-Culture

**Figure 6:**
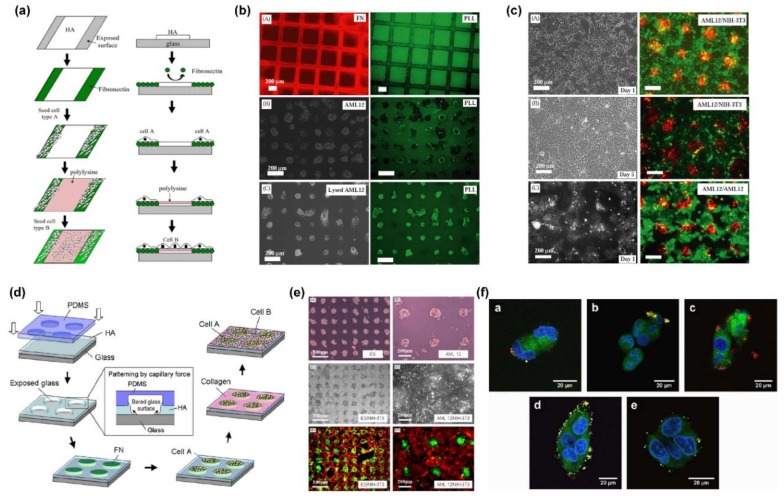
(**a**) Schematic diagram showing the layering approach to pattern cell co-cultures; (**b**) patterned hyaluronic acid surfaces attached with poly-l-lysine and immobilized cells; (**c**) patterned co-cultures of hepatocytes with fibroblasts; (**d**) schematic diagram of the fabrication of the co-culture system using LbL assembly technique; (**e**) patterned cell culture and co-culture on hyaluronic acid/collagen surface; (**f**) confocal laser scanning microscopy images of hepatocytes after co-culture on different chitosan/alginate LbL assembly films with nanoparticles. Reprinted from [99,100,101] with permissions from Elsevier.

The cell co-culture technique is of great importance in proof-of-principle studies, cell-cell interaction evaluation, and clinical transformation for various tissue engineering applications [97,98]. To achieve the complexity and organization of the in vivo cellular microenvironment, cell co-culture scaffolds with patterned coating and designed anisotropic properties are greatly needed, which can be effectively created using the LbL assembly technique due to its capacity of nanometer-scale engineering of hierarchical surfaces. Khademhosseini et al. reported an effective approach to prepare LbL assembly multilayers of hyaluronic acid and poly-l-lysine for patterned cell co-cultures, as presented in Figure 6a–c [99]. Based on the ionic adsorption of poly-l-lysine to hyaluronic patterns, the scaffold surface could be switched from cell repulsive to adherent, facilitating the adhesion of other type of cells. Furthermore, they examined the utility of this approach for co-culture patterns using different cell systems of hepatocytes or embryonic stem cells with fibroblasts. Micropatterned cell co-cultures using LbL deposition of ECM components were proposed by Fukuda et al. for creating effective tools for cell-cell interaction studies and tissue-engineering applications (Figure 6d,e) [100]. The co-culture scaffolds were coated with LbL assembly of ECM components (i.e., hyaluronic acid, fibronectin, and collagen) in which fibronectin was used to create cell-adhesive islands and the collagen was used to change the non-adherent hyaluronic acid pattern to cell adherent for the other cell type, respectively.

To achieve selective cell targeting, Zhou et al. prepared chitosan/alginate multilayer coatings on poly(lactide-co-glycolide) nanoparticles for antifouling protection and folic acid binding by LbL self-assembly (Figure 6f) [101]. They carried out cellular uptake measurements by co-culturing different cells, revealing a facile way to sequentially tailor nanoparticle surfaces to reduce unspecific interactions and to attach other molecules for successive cell selective adhesion. In this study, the nanoparticles were employed as substrates and endowed with selective targeting properties, fully indicating a promising design capability of nanodrugs using this strategy for targeted cell therapy. In addition, Kidambi et al. reported the fabrication of patterned cell co-cultures using LbL assembly of synthetic polymers without the aid of adhesive proteins/ligands [102]. As an alternative approach for co-culture scaffold fabrication, this strategy provides flexibility in design and development of cell-specific surfaces for tissue engineering.

## 5. LbL Self-Assembly of 3D Scaffolds for Tissue Engineering

### 5.1. 3D Scaffolds of ECM Films

The design of ECM multilayered scaffolds that resemble the hierarchical, lattice-like structure of tissues poses a big challenge for tissue engineering. Such ECM multilayer scaffolds can effectively regulate the interactions between different cells and function as natural tissues. Rajagopalan et al. described an effective approach to fabricate 3D scaffolds that mimic the micro-environment surrounding cells in vivo [103]. By using this strategy, different constructs composed of alternating layers of cells and biocompatible ECM scaffolds were fabricated, involving hepatocyte-scaffold-hepatocyte, hepatocyte-scaffold-endothelial cell, and hepatocyte-scaffold-fibroblast constructs, fully indicating the potential to generate constructs of various tissue types. Kim et al. developed the design of in vitro liver sinusoid mimics using LbL assembly scaffolds of chitosan and hyaluronic acid [104]. Silva et al. prepared nanostructured 3D constructs based on chitosan and chondroitin sulphate multilayers by combining LbL technology and template leaching for cartilage tissue engineering [50]. The obtained 3D scaffolds retrieved after paraffin leaching showed a high porosity and water uptake capacity of ~300%, and could maintain the chondrogenic phenotype and chondrogenic differentiation of multipotent bone marrow derived stromal cells, revealing the potential for clinical application in the field of cartilage tissue engineering. Although many studies have been carried out on 3D scaffolds of ECM films for tissue engineering, these studies were mainly focused on the material property investigation and in vitro studies; more detailed in vivo studies on 3D scaffolds of ECM films are still a big challenge and need to be carried out in the future.

### 5.2. 3D Scaffolds with Cell Composition Layers

**Figure 7:**
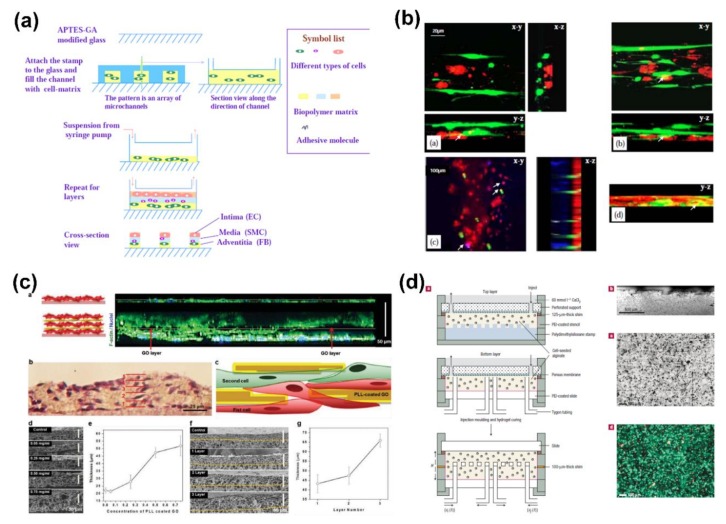
(**a**) Schematic illustration of the microfluidic LbL approach used to create 3D hierarchical systems with matrices and cells; (**b**) 3D images reconstituted from stacks demonstrate a two- or three-layer structure; (**c**) confocal cross-sectional images of the control group (top) and the 3 layer tissue constructs (bottom) after 2 days of culture. Hematoxylin and eosin stain images of 3 layer fibroblasts. Schematic illustration of the cross-section of the 2 layer construct. SEM images showing the cross-section and the thickness of 1, 2 and 3 layer constructs fabricated with various concentrations of poly-l-lysine-coated graphene oxide as interlayer films; (**d**) fabrication of cellular microfluidic scaffolds including the fabrication process and its resultant microstructures. Reprinted from [5,7,106] with permissions from Nature Publishing Group, Elsevier, and Wiley-VCH.

To achieve the ideal spatial distribution of cultured cells with the ECM micro-environment, various cells have been used as building blocks to construct 3D scaffolds for tissue engineering using LbL assembly. Compared to other LbL assembly methods, microfluidic LbL patterning is widely used due to its region-specific capacity. By using microfluidic LbL patterning of 3D biopolymer matrices, Tan et al. created biologically relevant cellular arrangements on scaffold surfaces, revealing a robust strategy to enhance cellular pattern integrity and effectively control cellular microenvironment, as shown in Figure 7a,b [7,47]. They investigated the effects of channel size, cell type, and matrix composition on pattern integrity. During their subsequent studies, the hierarchical biomimetic multilayer constructs with layers of cells and biopolymers in micro-channels were created for blood vessel engineering, indicating the controllable patterning of cells and ECM environments in 3D [105]. Besides biopolymers, carbon-based materials like graphene oxide were also used to combine with cells to form 3D tissue scaffolds. Shin et al. proposed an effective strategy to mimic the structure and function of native ECM materials using LbL assembly of cells separated with graphene oxide [106]. The resultant graphene oxide-based structures were used as adhesive sheets for cells and allowed the formation of multilayer cell constructs, showing great potential in engineering 3D tissues with enhanced organization and mechanical integrity, as displayed in Figure 7c.

Furthermore, certain structural designs and new LbL assembly techniques have been proposed to mimic the native and complex 3D cellular architecture for tissue engineering. Feng et al. described LbL seeding of smooth muscle cells in deep micro-channels to create aligned multilayers for vascular engineering [107]. Different from most other methods, Choi and co-workers proposed a new strategy to fabricate microfluidic structures within 3D cell-seeded scaffolds [5]. This approach could control the chemical environment on a micrometer scale within a macroscopic scaffold, as shown in Figure 7d. The resultant microfluidic channels allowed efficient distribution control and exchange of soluble chemicals, indicating a new format for the design and development for tissue engineering.

### 5.3. 3D Scaffolds with ECM Film Encapsulated Cells

**Figure 8:**
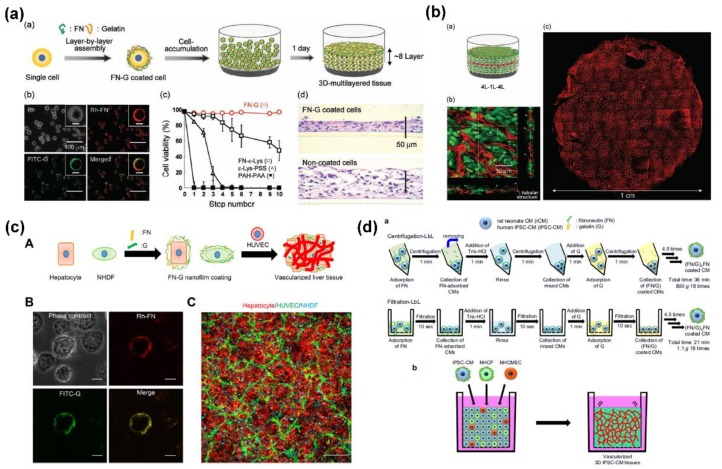
(**a**) Rapid construction of 3D multilayered tissues with endothelial tube networks by the cell-accumulation technique and the phase/fluorescent microscopic images, cell viability, and hematoxylin and eosin staining images of the resultant tissues; (**b**) schematic illustration, cross-section image, and reconstructed fluorescent image of 3D multilayered tissues; (**c**) the construction of vascularized liver tissue using LbL cell coating technique; (**d**) schematic illustrations of centrifugation-LbL and filtration-LbL for nanofilm coating on cell surfaces, and construction of vascularized 3D tissues by the cell accumulation technique. Reprinted from [112,113,114] with permissions from Wiley-VCH and Elsevier.

To create ideal 3D cell-polymer material composites for tissue engineering, the methodology to construct 3D multilayers composed of various cells with a nanometer-sized ECM layer is highly desired [108,109,110]. The principle of this design is that the formation of a nanometer-sized ECM layer on the cell surface first provides a cell-adhesive surface for the second type of cells. Due to the unique biocompatibility, ECM components of fibronectin and gelatin have been widely selected as building blocks to create nanometer-sized ECM films on different cell surfaces. Matsusaki et al. described the fabrication of well-organized cellular multilayers by constructing fibronectin-gelatin nanocoatings with designed thickness on cell surfaces using LbL assembly [111]. Furthermore, xenogeneic cellular multilayers such as blood vessels were constructed. By constructing fibronectin-gelatin coating nanofilms on a single cell, Nishiguchi et al. developed a cell-accumulation-based LbL assembly technique to rapidly construct 3D multilayered tissues with endothelial tube networks, as shown in Figure 8a,b [112]. Especially, the layer number, cell type, and location could be controlled by adjusting the seeding cell number and order using this technique. Recently, Sasaki et al. successfully constructed homogenous, dense, and vascularized liver tissue thereby presenting the functional ability of using the LbL cell coating technique (Figure 8c) [113]. This approach allowed loading of cells sterically onto other cells coated with LbL assembly of fibronectin and gelatin, and led to improved cellular function in terms of human albumin production and cytochrome P450 activity in vitro. Besides the conventional methods, a new filtration LbL assembly technique was introduced by Amano et al. to develop vascularized pluripotent stem cell-derived 3D-cardiomyocyte tissues for pharmaceutical assays, as shown in Figure 8d [114]. Benefiting from the integrated capacity of high yield and low damage, the constructed vascularized tissues could be a promising tool for tissue regeneration and drug development.

## 6. Concluding Remarks and Perspectives

LBL assembly, as a molecular-assembly technique, has been extensively used for the design and fabrication of biomaterial scaffolds for tissue engineering. The LbL assembly technique based on various driving forces and in the form of different technical strategies has been developed for a variety of biomedical applications, in particular, tissue engineering. To impart cell adhesive properties and biocompatibility to an existing substrate, to reassemble cells into specific tissues, or to function alone as tissue scaffolds, LbL assembly in the forms of multilayer nanofilms, scaffold nanocoatings, and 3D scaffolds have been created and served as new biomimetic matrices for tissue engineering. Yet important issues regarding LbL technologies, formation mechanisms, and applications, such as driving forces, regulation strategies, and biological functions still need further investigation. Here, we have reviewed the utilization of LbL assembly to design and create nanofilms, nanocoatings, and 3D scaffolds for tissue engineering.

In spite of remarkable progress in the development of LbL assembly biomaterials and related tissue engineering applications, some challenges still remain which may restrict their practical use. The challenges in the field of LbL assembly technique include the design and optimization of the technology parameters to obtain fast and stable coatings to enable long-term storage of multilayer systems. Moreover, the “black box” of LbL assembly techniques, focused on what materials are used (the input) for assembling the desired functional films (the output), has not yet been unpacked for specific tissue engineering applications. The current applications of LbL assembly in the field of tissue engineering are mainly focused on cell cultures in vitro, while the construction of complicated and functional tissues or organs and their practical applications in vivo still remain a great challenge. In addition, combinations between the LbL self-assembly technique and other multidisciplinary approaches should be explored to develop new LbL assembly biomaterials with multi-functions for tissue engineering. The potential for manufacture and application of LbL assembly biomaterials in the tissue engineering field is apparently unlimited, and we expect that continuous efforts in developing functional biomaterials using LbL assembly will address the current challenges and contribute to further advances in tissue engineering.
